# Thermal manipulation of plasmons in atomically thin films

**DOI:** 10.1038/s41377-020-0322-z

**Published:** 2020-05-18

**Authors:** Eduardo J. C. Dias, Renwen Yu, F. Javier García de Abajo

**Affiliations:** 1 ICFO-Institut de Ciencies Fotoniques, The Barcelona Institute of Science and Technology, 08860 Castelldefels (Barcelona), Spain; 2 ICREA-Institució Catalana de Recerca i Estudis Avançats, Passeig Lluís Companys 23, 08010 Barcelona, Spain

**Keywords:** Optoelectronic devices and components, Optoelectronic devices and components, Optical properties and devices, Nanocavities, Optical properties and devices

## Abstract

Nanoscale photothermal effects enable important applications in cancer therapy, imaging and catalysis. These effects also induce substantial changes in the optical response experienced by the probing light, thus suggesting their application in all-optical modulation. Here, we demonstrate the ability of graphene, thin metal films, and graphene/metal hybrid systems to undergo photothermal optical modulation with depths as large as >70% over a wide spectral range extending from the visible to the terahertz frequency domains. We envision the use of ultrafast pump laser pulses to raise the electron temperature of graphene during a picosecond timescale in which its mid-infrared plasmon resonances undergo dramatic shifts and broadenings, while visible and near-infrared plasmons in the neighboring metal films are severely attenuated by the presence of hot graphene electrons. Our study opens a promising avenue toward the active photothermal manipulation of the optical response in atomically thin materials with potential applications in ultrafast light modulation.

## Plasmonics: photothermal modulation

A new breed of ultrafast light modulators that operate across the visible to terahertz regions may be possible by harnessing photothermal control of plasmons in graphene and thin-metal films. Theoretical analysis performed by Eduardo Dias and coworkers from the ICFO research institute in Barcelona, Spain suggests that ultrafast optical excitation of thin gold/silver-graphene structures can significantly raise the electron temperature in the carbon layer and as a result dramatically modify the plasmonic response. Calculations indicate that strong changes (>70%) in the optical reflection of such structures are possible, with a strength that increases with the fluence of the excitation pulses. Importantly, the fluence is below the damage threshold of the materials. The photothermal modulation approach could have useful applications ranging from all-optical light modulation to optical sensing.

## Introduction

Heat generation driven by light absorption in nanostructures has proven useful for photothermal therapy^1–3^, nanoscale imaging^4,5^, data storage^6^, photocatalysis^7^ and photodetection^8,9^. These important applications have been extensively investigated in conducting materials due to the substantial enhancement produced in the strength of nanoscale photothermal processes as a result of the excitation of their collective electron oscillations, known as plasmons^10–12^. Among plasmonic materials, graphene offers additional appealing properties, such as large field confinement and enhancement^13–15^, remarkably low optical losses^14,16^ and the ability to tune its plasmons electrically^17–27^, which have generated expectations for applications in optical modulation^23,28–31^, light detection^8,17,22,32,33^, and sensing^34–36^. Unfortunately, plasmons in graphene have been observed only in the mid-infrared (mid-IR) spectral range, far from the technologically appealing visible and near-infrared (vis-NIR) spectral regime. Photothermal excitation has been proposed as a way to reach the vis-NIR plasmonic regime^37^, but experimental attempts in this direction have only been limited to the mid-IR^38,39^. As an alternative approach to extrapolate the appealing plasmonic properties of graphene to vis-NIR, both monolayer noble metals^40^ and hybrid systems formed by graphene in close proximity to few-nanometer metal films^41^ have been predicted to display relatively low losses and a large electrical tunability in the vis-NIR domain. Progress in this direction has been recently made with the experimental demonstration of plasmons in laterally patterned sub-2-nm crystalline silver^42^ and thicker amorphous gold^43^ films. In particular, epitaxially grown silver films serve as a novel platform for ultracompact nanophotonic devices that can further benefit from the comparatively high quality factor of plasmons in defect-free crystalline silver samples^42^.

Under ultrafast light irradiation, the absorbed energy is first deposited on conduction electrons, which remain at an elevated temperature for ~1 ps before transferring a substantial fraction of heat to the atomic lattice^44^. Importantly, due to the small electronic heat capacities of graphene^37,45^ and ultrathin noble metals^46^ compared with their total heat capacities, the lattice remains close to ambient temperature for optical pump-pulse fluences as high as ~10 J/m^2^, which is the upper limit of the range typically used to investigate thin-film dynamics through pump-probe spectroscopy^47,48^. In combination with the strong temperature dependence of the graphene optical conductivity, the transmission and reflection of a light probe can undergo radical variations produced by heating^49^, while intense photothermal effects should be expected due to the exceptionally small electronic heat capacity and generally weak electron-phonon coupling in this material^45^. Metals present a weaker thermal dependence, although the effects of intense pumping have also been observed to affect the probe as heat propagates away from the pumped region^48^. Consequently, graphene, thin metal films and hybrid systems comprising these materials hold strong potential to achieve large photothermal modulation over a broad spectral range extending from the visible to the THz region. Despite the vast range of applications that such modulation could open, the ultrafast photothermal response of these systems has been poorly explored.

In this paper, we theoretically investigate the thermal control of plasmons assisted by light absorption in graphene, thin metals and graphene/metal hybrid films. Based on a realistic description of the temperature-dependent optical properties of these materials, combined with the light-electron-lattice heat flow under ultrafast optical pumping, we predict a remarkable thermally driven attenuation of the plasmon strength in mid-IR graphene plasmons and vis-NIR metal plasmons of hybrid systems. This attenuation is directly revealed by plotting the reflection coefficient of planar structures. We further propose a practical scheme to exploit this dependence, consisting of laterally patterning the metal so that propagating external light can directly couple to the noted plasmons. Our results predict a large modulation of the light reflection produced by optical pumping using attainable intensities below the damage threshold level. Graphene plays an important role in these structures because in its absence the photothermal response is much weaker; we relate this result to the small heat capacity of the carbon layer. Our findings open a promising route towards ultrafast photothermal light modulation over a broad spectral range extending from vis-NIR to far-IR.

## Results

**Fig. 1:**
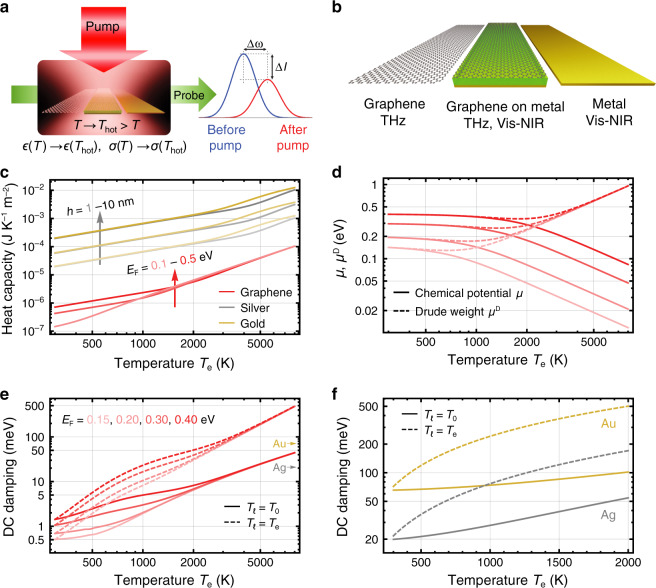
Thermo-optical properties of graphene and thin metal films. **a** Schematic representation of a thermoplasmonic light modulation system; a plasmon-supporting structure is optically pumped to generate a high electron temperature in an optical absorber placed inside it, thus inducing sizeable variations in the plasmon frequencies and intensities that are in turn translated into a large modulation of scattered probe light. **b** We consider thermoplasmonic systems combining graphene and thin metallic films, in which pumping light is preferentially absorbed by the former; the spectral operation range is indicated for each of these structures. **c** Dependence of the electronic heat capacity of graphene (calculated from Eq. (4) in Methods for different Fermi energies *E*_F_) and thin metallic films (obtained from ref. ^70^ for different thicknesses *h*) on electron temperature *T*_e_ when the lattice temperature ${T}_{\mathrm{\ell}}$ remains at the room value *T*_0_ = 300 K. **d** *T*_e_ dependence of the chemical potential *μ* (solid curves) and Drude weight *μ*^D^ (dashed curves) in graphene for different Fermi energies (see color code in **e**). **e**, **f** *T*_e_ dependence of the DC scattering rate in **e** graphene and **f** noble metals when the lattice temperature is either ${T}_{\mathrm{\ell}} = {T}_0$ (solid curves) or ${T}_{\mathrm{\ell}}\,=\,{T}_{\mathrm{e}}$ (dashed curves)

We illustrate the main concept explored in this paper in Fig. 1a. Although the manipulation of the graphene conductivity is possible through electrical gating or chemical doping, these methods cannot reach the ultrafast regime that might become necessary for future applications. We need to resort to intrinsically ultrafast approaches, such as optical pump-probe techniques. In the present work, we consider that an optical pump heats the electrons of a part of a structure, causing large changes in the optical response of the entire system, which in turn translate into frequency and intensity modulations of its optical resonances. We apply this idea to graphene, few-monolayer noble metal films and graphene/metal hybrid systems (Fig. 1b). Before analyzing the optical modes of these structures, it is useful to review the photothermal properties of their constituents, chosen to be among the best currently available plasmonic materials.

### Thermoplasmonic properties of atomically thin films

Graphene displays stronger photothermal effects than noble metals (see Fig. 1c) due to its conical band structure, in contrast to the free-electron-like behavior of the latter. A small amount of heat deposited on graphene electrons causes large temperature variations and changes in its optical response because the electronic heat capacity of this material is remarkably small^37^. This effect is thus an ideal ingredient for the pump-probe scenario envisioned in Fig. 1a. We anticipate that the strong graphene photothermal response can be exploited to modulate (vis-NIR) metal-like and (THz) acoustic plasmons in graphene/metal hybrid systems (Fig. 1b). The electronic heat capacity of graphene increases with the Fermi energy *E*_F_ (i.e., by injecting more electrons in the system, see Fig. 1c), but it still remains orders of magnitude lower than the lattice heat capacity (~350 JK^−1^m^−1^ at room temperature^50^) for realistic levels of doping, and the same is true for noble metals. A femtosecond optical pump can then raise the electron temperature *T*_e_ to 1000′s K, while heat transfer to the lattice, which takes place over a picosecond timescale^44^, does not substantially move the lattice temperature $T_{\mathrm{\ell}}$ away from the ambient level *T*_0_. In fact, we estimate that for fluences of the order of 1–5 mJ/m^2^, which roughly correspond to ultrafast pulses of ~100 fs with peak intensities of 0.1–0.5 GW/cm^2^, the electron temperature in graphene increases up to ~5000 K^51,52^, whereas electrons in the metal stay within a few 100′s K, as shown in Fig. S1 in the Supplementary Information (SI). This finding is a direct consequence of the much lower electronic heat capacity in graphene than in metals, as shown in Fig. 1c. We stress that the conditions described above are well below the reported damage threshold of graphene^53^. Therefore, in what follows, unless otherwise stated, we assume $T_{\mathrm{\ell}} = T_0 = 300\,{\mathrm{K}}$ in both graphene and metal, which is a good approximation under the conditions of typical pump-probe experiments during a short time after pumping.

The optical response of graphene is highly dependent on *T*_e_ through the chemical potential *μ*, the Drude weight *μ*^D^ and the inelastic scattering rate 1/*τ*, which enter its optical conductivity *σ*, as shown in Eq. (1) of the Methods section. The chemical potential *μ* decreases monotonically with increasing *T*_e_ at different Fermi levels, while the Drude weight *μ*^D^ increases dramatically after a slight initial decrease as the temperature is raised (see Fig. 1d). The nontrivial temperature dependence of *μ*^D^ defines the resonance position of graphene plasmons, acting as an effective doping from the optical viewpoint (i.e., more low-energy electron transitions become available and contribute to the plasmonic strength as the Fermi level is smeared out with increasing temperature). In contrast, the plasmon frequencies in noble metals exhibit a very mild dependence on the electron temperature (see Methods).

The inelastic scattering rate of graphene also depends strongly on *T*_e_, as shown in Fig. 1e within the DC limit for clean samples (see Methods). During the first picosecond after optical pumping, we typically have $T_{\mathrm{e}} \gg T_{\mathrm{\ell}}$ and $T_{\mathrm{\ell}} \approx T_0 = 300\,{\mathrm{K}}$; under these conditions, the inelastic decay rate takes the values shown by solid curves in Fig. 1e. Eventually, the electrons and the lattice thermalize to a common temperature $T_{\mathrm{\ell}} = T_{\mathrm{e}}$, a situation in which the scattering rate becomes larger as a consequence of stronger electron-phonon coupling (dashed curves in Fig. 1e). We also note that the scattering rate increases with *E*_F_ because the phase space for electron-phonon interactions is enlarged. Similar trends of the temperature dependence of the scattering rates are found in noble metals, as shown in Fig. 1f (see Methods).

**Fig. 2:**
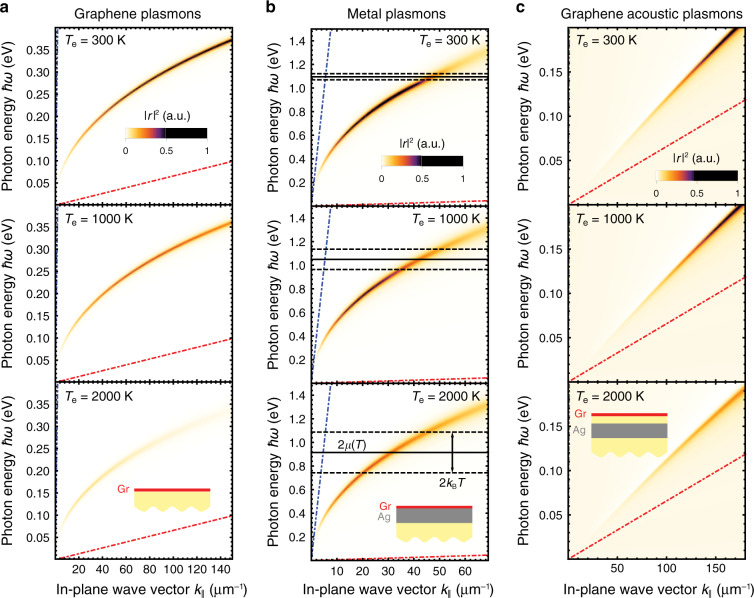
Temperature dependence of the plasmon dispersion relation. We plot the squared Fresnel reflection coefficient $\left| {r} \right|^2$ for p polarization as a function of the photon energy *ħω* and in-plane wave vector ${k}_\parallel$ for three different configurations: **a** graphene, **b** graphene/silver and **c** graphene/spacer/silver films, all of which are supported on a dielectric substrate (*ε* = 2), as shown in the lower insets. The silver layer thickness is 1 nm in **b** and 2 nm in **c** (i.e., approximately 4 and 8 (111) atomic layers, respectively^42^). The spacer in **c** has *ε* = 2 and 1-nm thickness. We consider three different values of the electron temperature *T*_e_ in graphene (top to bottom, see labels), while the metal electrons and the lattices of all materials are kept at room temperature *T*_0_ = 300 K. Graphene is doped to *E*_F_ = 0.55 eV in all cases. Light (${\omega} = {k}_\parallel {c}$, blue) and Fermi (${\omega} = {k}_\parallel {v}_{F}$, red) lines are shown for reference

In Fig. 2, we show the dispersion relations of three types of plasmonic modes supported by the structures sketched in the insets at three different graphene electron temperatures *T*_e_, as calculated using methods discussed in previous studies^37,41,54^. In all cases considered in Fig. 2, we find a strong thermoplasmonic modulation, showing that the plasmons become weaker at a higher *T*_e_ as a result of the increase in Re{*σ*}. For the mid-IR graphene plasmons considered in Fig. 2a, c, the thermoplasmonic modulation can be mainly attributed to an increase in the inelastic scattering rate when *T*_e_ rises (see Fig. 1e). We note that these types of plasmons are extremely subwavelength, deviating far away from the light line (blue broken lines, almost indistinguishable from the vertical axis) for increasing plasmon energies. In particular, acoustic plasmons, which are a special case of graphene plasmons that arise when a carbon sheet and a metal are brought in close proximity^55,56^, exhibit dispersion relations that are close to the onset of intraband electron-hole pair excitations in graphene (see Fig. 2c, red broken lines). Notably, the spacer thickness strongly affects the acoustic plasmon dispersion: in general, a thinner spacer produces shifts of the plasmon resonance towards lower frequencies, therefore resulting in more confined plasmons. In particular, spacer thicknesses down to 1 nm can be achieved using a few layers of exfoliated hexagonal boron nitride (hBN), while even a spacer consisting of a single hBN layer (roughly 0.4 nm) has been reported in the literature^56^. More information regarding the dependence of the acoustic plasmon dispersion on the spacer thickness and Fermi level is presented in Fig. S2 of SI. In contrast, metal plasmons (Fig. 2b), although still very subwavelength, are less confined; at low temperatures (Fig. 2b, top), they are quenched by coupling to interband transitions at photon energies above 2*E*_F_ (solid horizontal line), while thermal smearing of Dirac fermions shifts down the chemical potential *μ* for the *T*_e_ values under consideration and diffuses the quenching within an energy interval of roughly ±*k*_B_*T*_e_ (dashed horizontal lines) around 2*μ* (solid horizontal lines).

### All-optical manipulation of plasmons in planar structures

**Fig. 3:**
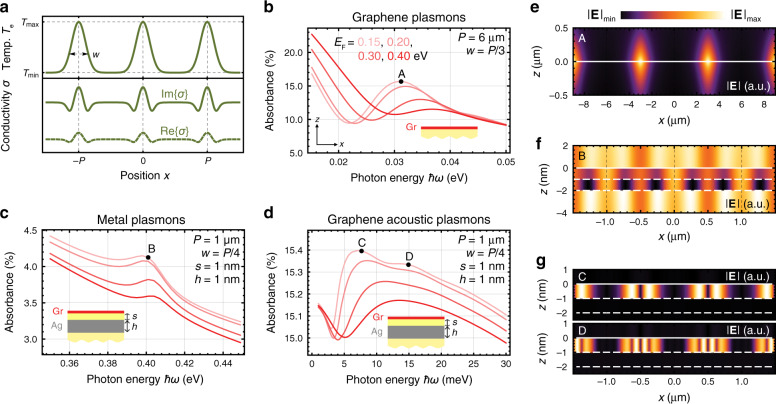
Optical modulation of plasmons in graphene and graphene/metal hybrid systems. We consider different structures (see insets) with equivalent spatial modulations of the electron temperature, optically imprinted on graphene (panel **a**, top) and consisting of a series of Gaussian profiles arranged with period *P*, FWHM *w* and peak temperature *T*_max_ = 8000 K, superimposed on a room temperature background *T*_min_ = 300 K; the resulting modulation of the conductivity is shown in the lower plot. **b**–**d** Absorbance spectra of two different structures with geometrical parameters *P*, *w, h* and spacings *s* as indicated by the labels; all dielectrics in these structures (yellow regions in the insets) are taken to have a permittivity *ε* = 2; the graphene Fermi energy is varied in all plots according to the legend in **b**; spectra reveal pure graphene plasmons in **b**, graphene-modulated metal-like plasmons in **c** and graphene acoustic plasmons in **d**. **e**–**g** Electric field distributions in the structures of **b**–**d** at the plasmon frequencies indicated by the corresponding spectral points A–D; solid and dashed white lines indicate the graphene and metal interfaces

Because plasmon resonances are deep subwavelength excitations (see Fig. 2), extra momentum is required to couple external light into them, typically provided by lateral patterning (e.g., carving gratings or imprinting surface modulations^57^) or additional elements (e.g., sharp tips in scanning near-field optical microscopes). Recently, an alternative route to excite graphene plasmons has been proposed^49^, where two interfering optical pump beams create a periodic spatial temperature profile in graphene to assist the coupling of a probe beam to plasmons. The characteristic diffusion time of the temperature profile created by the optical pump is of the order of a few hundred femtoseconds^58^, which should allow a probed optical pulse to excite plasmons, as considered in this study. Here, we further demonstrate that not only graphene plasmons but also metal plasmons or even subwavelength acoustic plasmons can be excited using this approach. We assume a periodic pattern of electron temperature *T*_e_ created by the optical grating pump only inside the graphene sheet with a modulated temperature range extending from 300 to 8000 K. Due to the strong temperature dependence of the graphene conductivity *σ* (see Fig. 1), this is spatially modulated and serves as an effective grating (see Fig. 3a). We simulate this type of periodic system using a previously described numerical procedure^55^.

Applying this method to a single extended graphene sheet supported on an *ε* = 2 substrate, we indeed find graphene plasmons in the absorbance spectra of a light probe (Fig. 3b), which are more pronounced for lower Fermi energies due to the stronger spatial variation in the graphene conductivity (see Fig. S3a in SI). The corresponding resonant near-field distribution (Fig. 3e) reveals plasmon confinement around the minima of *T*_e_, where Re{*σ*} is also minimum (see Fig. S3a). The presence of clear plasmonic resonances even in a pattern with such high temperatures shows that the larger losses experienced by plasmons at higher temperatures are not critical, as long as the lower part of the temperature modulation, where the plasmons are located, is sufficiently deep.

When graphene sits on top of a thin silver film separated by a thin dielectric spacer (see insets in Fig. 3c, d), the hybrid system can support both metal-like and acoustic plasmons. In this configuration, the probing light can still be scattered by the spatial modulation of the graphene conductivity, and therefore, for appropriate photon energies, it can excite those types of plasmons. When exploring the NIR optical response of this hybrid structure, we find absorption peaks associated with metal plasmons, in which the near-field distribution penetrates significantly into the metal film (see Fig. 3f). As a result, we find that 3.36% and 0.76% of the probe light are absorbed at the spectral peak B by metal and graphene layers, respectively. In addition, multiple acoustic plasmons can be excited in the same hybrid system in the THz domain, as shown in Fig. 3d. We plot the near-field distributions of the first two of them in Fig. 3g, where the plasmon energy is observed to be transversally confined inside the dielectric spacer but also laterally concentrated around the regions of minimum Re{*σ*} (see Fig. 3a), with a larger number of nodes along the horizontal direction for higher energy modes, similar to classical standing waves of increasing order in a confining potential. At the first acoustic plasmon resonance (peak C), 15.09% and 0.33% of the probe light are absorbed by the metal and graphene, respectively. The larger absorption observed for acoustic plasmons is presumably due to their tighter vertical confinement, which maximizes the fraction of the mode energy present in the metal.

### Thermal modulation of ultrathin structured films

**Fig. 4:**
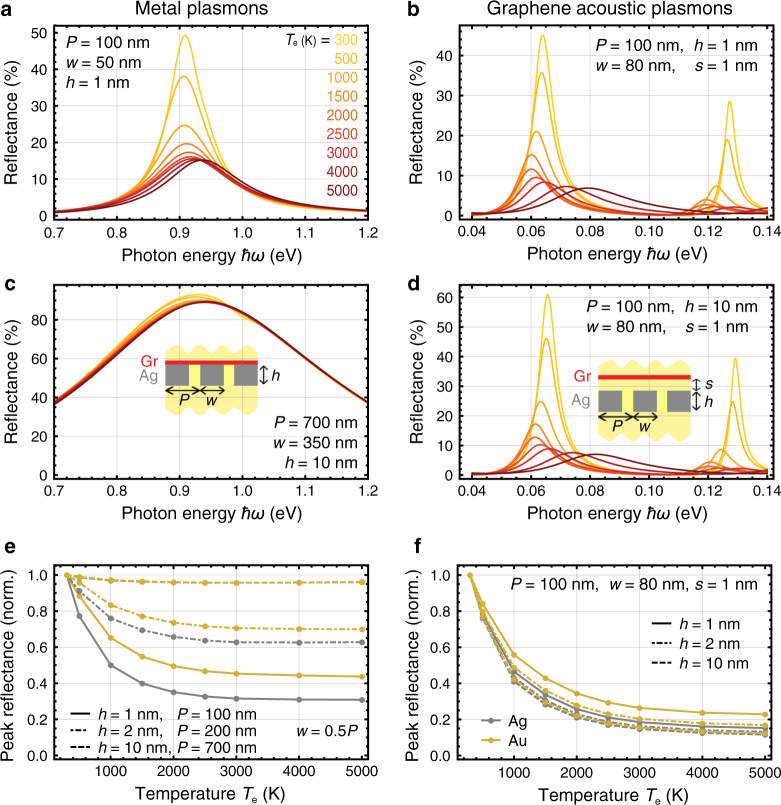
Thermal modulation of plasmons in hybrid graphene/structured-metal systems. **a**–**d** Variation in the reflection spectra of graphene/silver gratings in the **a**, **c** NIR and **b**, **d** far/mid-IR plasmonic regions for different graphene electron temperatures *T*_e_; the silver thickness is 1 nm in **a**, **b** and 10 nm in **c**, **d**. **e**, **f** Peak reflectance as a function of the graphene electron temperature for structures with different types of metal and metal thicknesses (see geometrical parameters indicated by labels). In **a**, **c**, **e**, plasmons are metal-like, and graphene is doped to *E*_F_ = 0.55 eV. In **b**, **d**, **f**, plasmons are acoustic, and graphene is doped to *E*_F_ = 0.4 eV. All structures are embedded in an *ε* = 2 dielectri

Lateral patterning of the structures considered above directly enables light coupling to their plasmons. In Fig. 4 and S4, we study the thermo-optical properties of these types of structures by considering a thin metal grating placed underneath a graphene sheet. The spectral positions of the plasmons sampled by external light are then controlled by the grating period and metal thickness.

We first investigate metal-like plasmons occurring at a higher energy and supported primarily by the metal. In Fig. 4a, we consider graphene doped to a Fermi energy *E*_F_ = 0.55 eV and a silver grating period chosen such that the plasmon energy is slightly smaller than 2*E*_F_, so that a large modulation is expected by thermally activating interband transitions within a spectral region of size ±*k*_B_*T*_e_ around 2*E*_F_, similar to the results presented in Fig. 2. At room temperature, 2*μ* ≈ 2*E*_F_ is above the plasmon energy, so we have well-defined modes with a quality factor ~12.5 observed in the reflection spectrum of the patterned 1-nm silver film (see *T*_e_ = 300 K curve in Fig. 4a). However, when graphene electrons are heated to a high temperature via ultrafast optical pumping, the chemical potential can be reduced (Fig. 1d), enabling Landau damping of the plasmon, whose quality factor drops to 6.3 at *T*_e_ = 5000 K (see Fig. 4a). In contrast, for a 10-nm-thick silver ribbon array, plasmons are already substantially broader at room temperature (see Fig. 4c), presumably as a result of larger radiative losses^42^, while we observe weaker thermoplasmonic modulation due in part to the smaller fraction of plasmon energy overlapping the lossy graphene region as the film is made thicker. The peak reflectance at the NIR plasmon resonance of gold and silver gratings in the hybrid structures under consideration is summarized in Fig. 4e as a function of *T*_e_ for different metal thicknesses. The modulation depth, which is larger for silver and increases in thinner metal films, takes values as large as ~70% for the 1-nm-thick silver structure. These results demonstrate that the thermo-optical response of graphene allows us to reach similar levels of NIR light modulation in neighboring metal plasmons, as previously predicted by exploiting the electro-optical response of this material^41^.

The addition of a 1-nm-thick dielectric spacer between graphene and the thin silver grating enables pronounced acoustic plasmon resonances in the THz range at *T*_e_ = 300 K, where both the first-order and second-order modes are observed in the reflection spectra (Fig. 4b). Similarly, at the elevated electron temperatures induced by optical pumping, those resonances are damped, driving a change in the quality factor from 7.8 at *T*_e_ = 300 K to 2.6 at *T*_e_ = 5000 K in the first-order resonance. In contrast to metal plasmons, the mode energies in this regime are far from the interband transition region, thus preventing Landau damping; plasmon damping is instead mediated by the increase in the inelastic scattering rate with temperature (see Fig. 1e). In addition, the nontrivial spectral shift of the plasmon resonance with varying *T*_e_ closely follows the temperature behavior of the Drude weight shown in Fig. 1d. It should be noted that, unlike metal plasmons, there is not a strong dependence on the metal thickness (cf. Fig. 4b, d) because the acoustic plasmon energy is mainly concentrated inside the dielectric spacer (see Fig. 3g), and a 1-nm metal film already produces nearly maximum confinement^59^. In fact, the extended study of the modulation depth of acoustic plasmons presented in Fig. 4f indicates a remarkable maximum value of ~80%, independent of both thickness and type of metal.

**Fig. 5:**
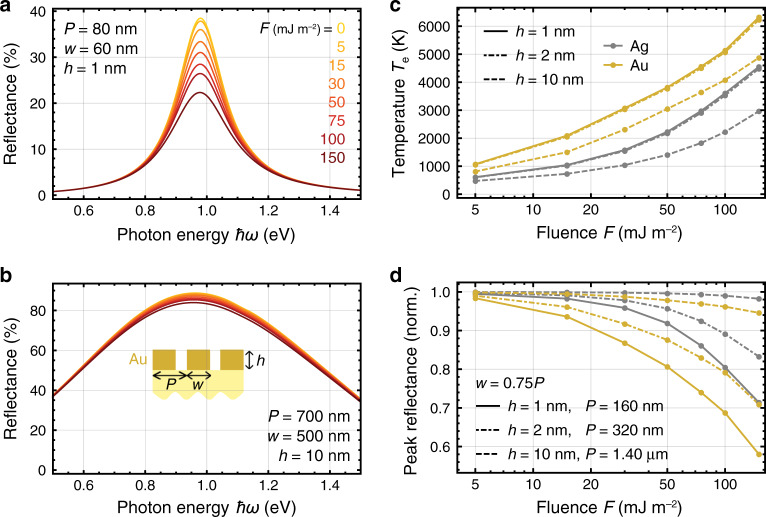
Thermal modulation of metal plasmons. **a**, **b** Variation in the probed reflection spectra in a gold ribbon array (see inset in **b**) under different pump fluences at 785 nm wavelength; the gold thickness is 1 nm in **a** and 10 nm in **b**. **c** Fluence dependence of the electron temperature of extended gold and silver films of different thicknesses immediately after optical pumping. **d** Peak reflectance as a function of the pump fluence for different metal types and thicknesses, normalized to the value at zero fluence with geometrical parameters as indicated by labels. The ribbon array is placed on an *ε* = 2 dielectric substate in all cases

We finally explore in Fig. 5 (see also Fig. S5 in SI) the modulation that can be reached in metallic gratings without any graphene, for which we have to adjust the pump fluence in order to heat the metal electrons to a sufficiently high temperature. Thermo-optical modulation is then produced due to the increase in the inelastic scattering rate with temperature (see Fig. 1f). The reflection spectra plotted in Fig. 5a show a clear variation in the quality factor for a 1-nm-thick gold ribbon array as the pump fluence is increased. Specifically, the quality factor drops from 5.3 to 4.0 when the fluence varies in the 0–150 mJ/m^2^ range. Because the temperature elevation is smaller in thicker metal structures (see Fig. 5c and Fig. S1 in SI), we expect a weaker temperature dependence in the reflection spectra, which is corroborated in Fig. 5b for a thickness of 10 nm. We summarize the relative variation in the peak reflectance as a function of pump fluence for different materials and metal thicknesses in Fig. 5d. In contrast to the hybrid graphene/metal structures, we now observe a larger modulation with gold than with silver. In particular, a maximum modulation depth of ~10% (~30%) is predicted for a pump fluence of 20 mJ/m^2^ (100 mJ/m^2^) in 1-nm-thick (four atomic layers) gold gratings, which is a smaller value than in hybrid structures, despite the large fluences under consideration compared with those needed to heat graphene electrons to the values of *T*_e_ used in Fig. 4.

## Discussion

In summary, the remarkably small electronic heat capacity of atomically thin systems, such as graphene and few-atomic-layer noble metal films, allows us to elevate their electron temperature via ultrafast optical pumping in such a way that their optical responses are dramatically modified with moderate pump intensities below the damage threshold. This effect, which constitutes the basis for ultrafast thermo-optical modulation, is particularly strong in graphene due to the large photothermal response of this material, in which the conical electronic band structure causes large changes in the optical behavior for moderate amounts of deposited electronic heat. The photothermal modulation of the frequency and strength of graphene plasmons has been extensively studied in the past^37^, but their applicability has been limited to the mid-IR regime. We have shown that the addition of a noble metal film in close proximity to graphene enables similar levels of modulation >70% to be reached over a wider spectral range extending down to the visible domain. More precisely, optical pumping can bring graphene electrons to a high temperature, while the large heat capacity of the metal prevents similar heating inside it; then, high-energy metal-like plasmons are strongly affected by the opening of additional loss channels (i.e., formerly forbidden electron-hole pair transitions in the hot graphene electron gas); additionally, lower-frequency acoustic plasmons supported in these hybrid structures also become more lossy. A practical way to exploit these effects consists in laterally patterning the metal (e.g., into ribbons), thus breaking the energy-momentum mismatch that otherwise prevents light coupling into the plasmons of planar films. We envision applications in ultrafast optical modulation, which could be potentially pushed to the single-photon level through plasmon blockade in sufficiently small graphene nanostructures^60^. Spectrally resolved optical sensing could also be carried out by exploiting the temperature-dependent plasmon shifts reported here, which would enable a continuous sweep of the probe frequency as the system cools down over a picosecond timescale. Although the achieved thermally-induced spectral shifts can be limited by the presence of a finite diffusion time in the temperature profile, the amplitudes of the associated changes in the reflectivity provide an alternative sizeable signature for optical sensing in the ~300–3000 K temperature range.

## Methods

### Graphene conductivity

We use the local random-phase approximation to calculate the optical conductivity of graphene as^61,62^

1 $$\sigma \left( \omega \right) = \frac{{e^2}}{{\pi \hbar ^2}}\frac{{\mathrm{i}}}{{\omega + {\mathrm{i/}}\tau }}\left[ {\mu ^{\mathrm{D}} - {\int_0^\infty} {{\mathrm{d}}E\frac{{f_{\mu ,T_{\mathrm{e}}}\left( E \right) - f_{\mu ,T_{\mathrm{e}}}\left( { - E} \right)}}{{1 - 4E^2/\left[ {{\hbar}^2\left( {\omega + {\mathrm{i/}}\tau } \right)^2} \right]}}} } \right]$$

where *τ* is a phenomenological inelastic scattering lifetime, $f_{\mu ,T_{\mathrm{e}}}(E) = \left[ {{\mathrm{e}}^{\left( {E - \mu } \right)/k_{\mathrm{B}}T_{\mathrm{e}}} + 1} \right]^{ - 1}$ is the Fermi-Dirac distribution at an electron temperature *T*_e_, energy *E* and chemical potential *μ*, *k*_B_ is the Boltzmann constant and $\mu ^{\mathrm{D}} = \mu + 2k_{\mathrm{B}}T_{\mathrm{e}}\log \left( {1 + {\mathrm{e}}^{ - \mu /k_{\mathrm{B}}T_{\mathrm{e}}}} \right)$ is the Drude weight. The temperature-corrected chemical potential can be well approximated by^63^

2 $$\mu \approx \sqrt {\sqrt {E_{\mathrm{F}}^4 + \left( {2\log ^24} \right)^2\left( {k_{\mathrm{B}}T_{\mathrm{e}}} \right)^4} - 2\log ^24\left( {k_{\mathrm{B}}T_{\mathrm{e}}} \right)^2}$$

where *E*_F_ is the Fermi energy defined at zero temperature. The graphene conductivity has two contributions corresponding to the electronic intraband (first term in Eq. (1), proportional to *μ*^D^) and interband (integral term in Eq. (1)) transitions. Pauli blocking forbids interband transitions with energy below 2*E*_F_ at zero temperature, implying that the intraband response dominates at low temperatures and photon energies, where Re{*σ*} is roughly proportional to 1/*τ* (i.e., the intrinsic optical losses). However, at higher electron temperatures, the chemical potential becomes increasingly smaller than *E*_F_ (Fig. 1d), allowing transitions to take place at increasingly lower energies and therefore enhancing Re{*σ*}.

In general, the inelastic scattering rate 1/*τ* depends on both electron and lattice temperatures. For clean graphene samples (e.g., encapsulated graphene), the inelastic scattering rate is dominated by electron-phonon scattering. Following previous studies^64,65^, the energy-dependent scattering rate associated with the *b*-type phonon is given to first order by

$$\frac{1}{{\tau _b(\varepsilon _{\mathbf{k}}^l)}} = \mathop {\sum }\limits_{{\mathbf{k}}^\prime ,l^\prime } P_{{\mathbf{kk}}^\prime ,b}^{ll^\prime }\frac{{1 - f_{\mu ,T_{\mathrm{e}}}\left( {E_{{\mathbf{k}}^\prime }^{l^\prime }} \right)}}{{1 - f_{\mu ,T_{\mathrm{e}}}\left( {E_{\mathbf{k}}^l} \right)}}(1 - \cos \theta _{{\mathbf{kk}}\prime })$$

where θ_**kk′**_ is the scattering angle between incoming and outgoing wave vectors **k** and **k′**, $E_{\mathbf{k}}^l = l\hbar v_{\mathrm{F}}\left| {\mathbf{k}} \right|$ denotes the electron (*l* = 1) and hole (*l* = −1) energies in the conical band dispersion regime, *v*_F _≈ 10^6^ ms^–1^ is the Fermi velocity and

$$P_{{\mathbf{kk}}^{\prime},b}^{ll^{\prime}} = \frac{{2\pi }}{\hbar }\left| {g_{{\mathbf{kk}}^{\prime},b}^{ll^{\prime}}} \right|^2\left\{ {n\left( {\omega _{{\mathbf{q}},b}} \right)\delta \left( {E_{\mathbf{k}^\prime}^{l^{\prime}} - E_{\mathbf{k}}^l - \hbar \omega _{{\mathbf{q}},b}} \right) + \left[ {n\left( {\omega _{{\mathbf{q}},b}} \right) + 1} \right]\delta \left( {E_{\mathbf{k}^\prime}^{l^{\prime}} - E_{\mathbf{k}}^l + \hbar \omega _{{\mathbf{q}},b}} \right)} \right\}$$

is the electron-*b*-phonon scattering probability. Here, **q** = **k′−k**, $n\left( \omega \right) = \left[ {{\mathrm{e}}^{\hbar \omega /k_{\mathrm{B}}T_{\mathrm{\ell}} } - 1} \right]^{ - 1}$ is the Bose-Einstein distribution, $T_{\mathrm{\ell}}$ is the lattice temperature and $g_{{\mathbf{kk}}^\prime ,b}^{ll^\prime }$ are matrix elements for the *b*-type phonon that can be readily evaluated using Eqs. (16–21) and Table IV of ref. ^64^. Then, the total DC inelastic scattering rate is given by

3 $$\frac{1}{\tau} = \frac{{{\int} {\mathrm{d}}{\mathit{E}}\,D({\mathit{E})\partial f_{\mu,{\mathit{T}}_{\mathrm{e}}}\left( {\mathit{E}} \right)/\partial {\mathit{E}}} }}{{{\int} {\mathrm{d}}{\mathit{E}}\,D({\mathit{E}})\left[{\mathop{\sum}\nolimits_{\mathit{b}} \tau_{\mathit{b}}^{-1}\left( {\mathit{E}} \right)} \right]^{-1}\partial f_{\mu ,{\mathit{T}}_{\mathrm{e}}}({\mathit{E}})/\partial {\mathit{E}}}}$$

where $D(E) = 2|E|/\pi \hbar ^2v_{\mathrm{F}}^2$ is the electron density of states in graphene, including spin and valley degeneracy. We present in Fig. 1e the total inelastic scattering rate obtained from Eq. (3).

### Graphene heat capacity

The specific heat capacity of graphene (Fig. 1c) is calculated from the slope of the *T*_e_-dependent electronic heat as^63^

4 $$c_{\mathrm{e}} = \frac{\partial }{{\partial T_{\mathrm{e}}}}\left[ {\beta \frac{{\left( {k_{\mathrm{B}}T_{\mathrm{e}}} \right)^3}}{{\left( {\hbar v_{\mathrm{F}}} \right)^2}}} \right]$$

where

5 $$\beta = \frac{2}{\pi }\left[ {\mathop {\int }\nolimits_0^\infty x^2{\mathrm{d}}x\left( {\frac{1}{{{\mathrm{e}}^{x + \mu /k_{\mathrm{B}}T_{\mathrm{e}}} + 1}} + \frac{1}{{{\mathrm{e}}^{x - \mu /k_{\mathrm{B}}T_{\mathrm{e}}} + 1}}} \right) - \frac{1}{3}\left( {\frac{{E_{\mathrm{F}}}}{{k_{\mathrm{B}}T_{\mathrm{e}}}}} \right)^3} \right]$$

is a thermal coefficient.

### Temperature-dependent permittivity of noble metals

For ultrathin metal films, a recent study shows that the Drude response works extremely well compared with the random-phase approximation incorporating film electron wave functions and atomic-plane corrugation^59^. In addition, the Drude model is in agreement with recent observations of plasmons supported by few-atomic-layer crystalline silver films^42^. We therefore use the Drude model and write the metal permittivity as^48^

6 $${\it{\epsilon }}_{\mathrm{m}}\left( {\omega ,T_{\mathrm{e}},T_{\mathrm{\ell}} } \right) = {\it{\epsilon }}_\infty - \frac{{\omega^2_{\mathrm{p}}(T_{\mathrm{\ell}} )}}{{\omega \left[ {\omega + {\mathrm{i}}\gamma _{\mathrm{m}}(T_{\mathrm{e}},T_{\mathrm{\ell}} )} \right]}}$$

where *ε*_∞_ is a background accounting for the polarization of interband transitions, the plasmon frequency $\omega _{\mathrm{p}}(T_{\mathrm{\ell}} ) = \omega _{\mathrm{p}}(T_0)/\sqrt {1 + B(T_{\mathrm{\ell}} - T_0)}$ depends on lattice temperature $T_{\mathrm{\ell}}$ through the thermal expansion coefficient *B* (see Table 1), and the phenomenological damping rate $\gamma _{\mathrm{m}}(T_{\mathrm{e}},T_{\mathrm{\ell}} ) = \gamma _{\mathrm{m}}^{{\mathrm{e}} - {\mathrm{e}}}(T_{\mathrm{e}}) + \gamma _{\mathrm{m}}^{{\mathrm{e}} - {\mathrm{ph}}}(T_{\mathrm{\ell}} )$ is the sum of the temperature-dependent electron-electron and electron-phonon contributions. More precisely^66–68^,

7 $$\gamma _{\mathrm{m}}^{{\mathrm{e}} - {\mathrm{e}}}\left( {T_{\mathrm{e}}} \right) = \frac{{\pi ^3{{\Gamma \Delta }}}}{{12\hbar E_{\mathrm{F}}}}k_{\mathrm{B}}^2T_{\mathrm{e}}^2$$

(see Table 1 for values of the Fermi energy *E*_F_ and scattering coefficients Γ and Δ) and^67,68^

8 $$\gamma_{\mathrm{m}}^{{\mathrm{e}} - {\mathrm{ph}}}\left( {T_{\mathrm{\ell}} } \right) = \gamma_{0}\left( {\frac{2}{5} + \frac{{4T_{\mathrm{\ell}}^{5}}}{{\theta_{\mathrm{D}}^{5}}}\mathop{\int }\nolimits_{0}^{\theta_{\mathrm{D}}/T_{\mathrm{\ell}}} \frac{{z^{4}{d}z}}{{{\mathrm{e}}^{z} - 1}}} \right) \approx \gamma_{0}\frac{{T_{\mathrm{\ell}}}}{{\theta_{\mathrm{D}}}}$$

**Table 1:** Parameters used in Eqs. (6), (7) and (8) to calculate the temperature-dependent dielectric function of gold and silver, along with source references

Metal	*ε*_∞_^69^	*ħω*_p_(*T*_0_) (eV)^69^	*B* (K^−1^)^71^	*E*_F_ (eV)^46^	Γ^66^	Δ^66^	θ_D_ (K)^72^	*ħγ*_0_ (meV)
Au	9.5	9.06	14.2 × 10^−6^	5.53	0.55	0.77	170	39.5
Ag	4.0	9.17	19.7 × 10^−6^	5.49	0.55	0.73	215	14.5

where *γ*_0_ is found by matching the total inelastic scattering rate *γ*_m_(*T*_0_,*T*_0_) to the experimental results at ambient temperature *T*_0_ = 300 K (i.e., *ħγ*_m_ = 71 meV and 21 meV for gold and silver, respectively^69^; see Table 1 for the values of *γ*_0_ and the Debye temperature *θ*_D_). The rightmost approximate expression in Eq. (8) is valid in the $T_{\mathrm{\ell}} \gg \theta _{\mathrm{D}}$ limit. We plot *γ*_m_ in Fig. 1f for Au and Ag with different temperature combinations. We further use Eq. (6) to calculate the optical spectra shown in Fig. 5. We emphasize that the *T*_e_ dependence of the plasmon resonance frequency of metallic nanostructures is mostly controlled by that of the classical metal plasma frequency *ω*_p_, which is robust against the electron temperature range considered in this work (see Fig. 5 and Fig. S5 in SI).

## Supplementary information

Supplementary Information
