# Supplementary material for: Thermal manipulation of plasmons in atomically thin films
Source: Light Sci Appl. 2020 May 18;9:87. doi: 10.1038/s41377-020-0322-z (PMC7235028; doi:10.1038/s41377-020-0322-z)
Supplement: Supplementary file 1 — Supplementary Information [file 41377_2020_322_MOESM1_ESM.pdf]

Thermal Manipulation of Plasmons in Atomically Thin Films

– SUPPLEMENTARY INFORMATION –

Eduardo J. C. Dias,[1] Renwen Yu,[1,*] and F. Javier García de Abajo[1,2,†]

[1]ICFO-Institut de Ciencies Fotoniques,
The Barcelona Institute of Science and Technology, 08860 Castelldefels (Barcelona), Spain
[2]ICREA-Institució Catalana de Recerca i Estudis Avançats,
Passeig Lluís Companys 23, 08010 Barcelona, Spain

(Dated: April 13, 2020)

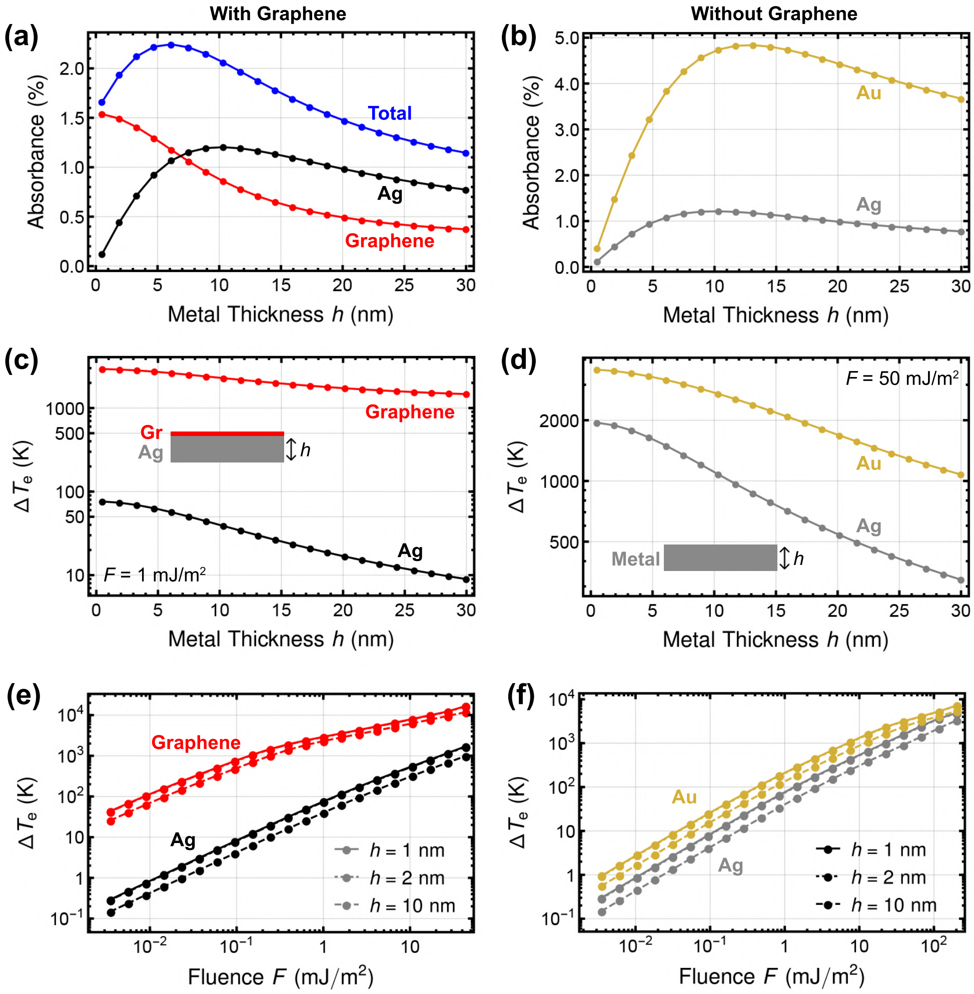

FIG. S1: Light absorbance and electron temperature increase in ultrathin graphene/metal and pure metal films. (a) Total absorbance calculated for the self-standing graphene/silver hybrid film depicted in the inset of (c) as a function of silver layer thickness. We also show partial contributions due to metal and graphene. The graphene is doped to $E_F = 0.5\,$eV. (b) Absorbance of self-standing gold and silver films as a function thickness. (c,d) Electron temperature increase ΔT_e in graphene and metal for the films considered in (a) and (b), respectively, as a function of metal thickness for the indicated pump fluences. (e,f) Electron temperature increase ΔT_e in the graphene and metal for the films considered in (a,b) as a function of the pump fluence for the indicated metal thicknesses. We consider a light wavelength of 785 nm in all panels.

*Corresponding author: renwen.yu@icloud.com
†Corresponding author: javier.garciadeabajo@nanophotonics.es

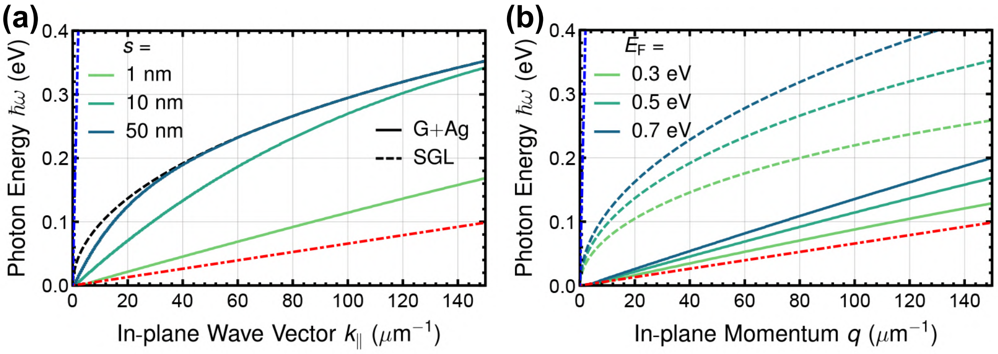

FIG. S2: Variation of the dispersion relation of graphene acoustic plasmons (solid curves) in the configuration of Fig. 2(c) (i.e., substrate/Ag/spacer/graphene/air) with either (a) spacer thickness s for fixed graphene Fermi energy $E_{\rm F} = 0.5\,$eV or (b) $E_{\rm F}$ for fixed $s = 1\,$nm. We show the dispersion relation without Ag (dashed curves), the light line ($\omega = ck_\parallel$, blue lines), and the Fermi line ($\omega = v_{\rm F} k_\parallel$, red lines) for reference. The Ag thickness is 1 nm, while the spacer and substrate permittivity is $\epsilon = 2$.

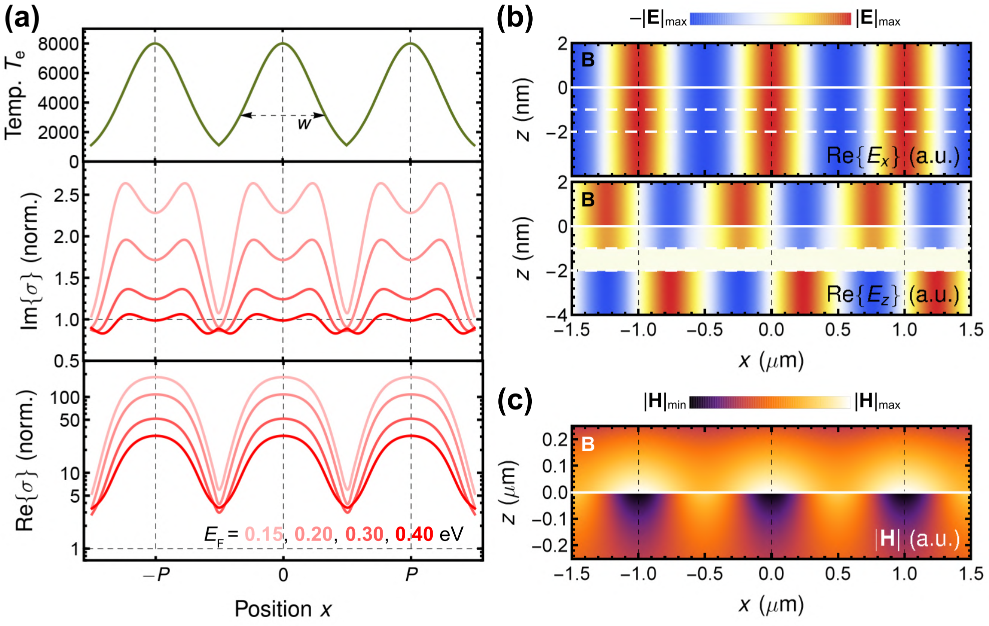

FIG. S3: All-optical modulation of plasmons in graphene/metal hybrid films. (a) Temperature profile used to obtain the results shown in Fig. 3(b) of the main text (top), and corresponding spatial modulation of the imaginary (middle) and real (bottom) parts of the graphene conductivity for different Fermi energies. The conductivity is normalized to its value at $T_0 = 300\,$K and calculated at the corresponding resonance frequencies shown in Fig. 3(b) of the main text for each Fermi energy. (b) Spatial distributions of the electric near-field components Re$\{E_x\}$ and Re$\{E_z\}$ for the spectral position B shown in Fig. 3(c) of the main text in the hybrid film formed by graphene (solid white line) and silver (region in between dashed white lines). (c) Spatial distribution of the magnetic field under the same conditions as in (b), calculated for a larger range of the out-of-plane coordinate z such that field oscillations due to excitation of the metal plasmon are clearly discernible. The thickness of the film (white line) cannot be resolved on the scale of the plot.

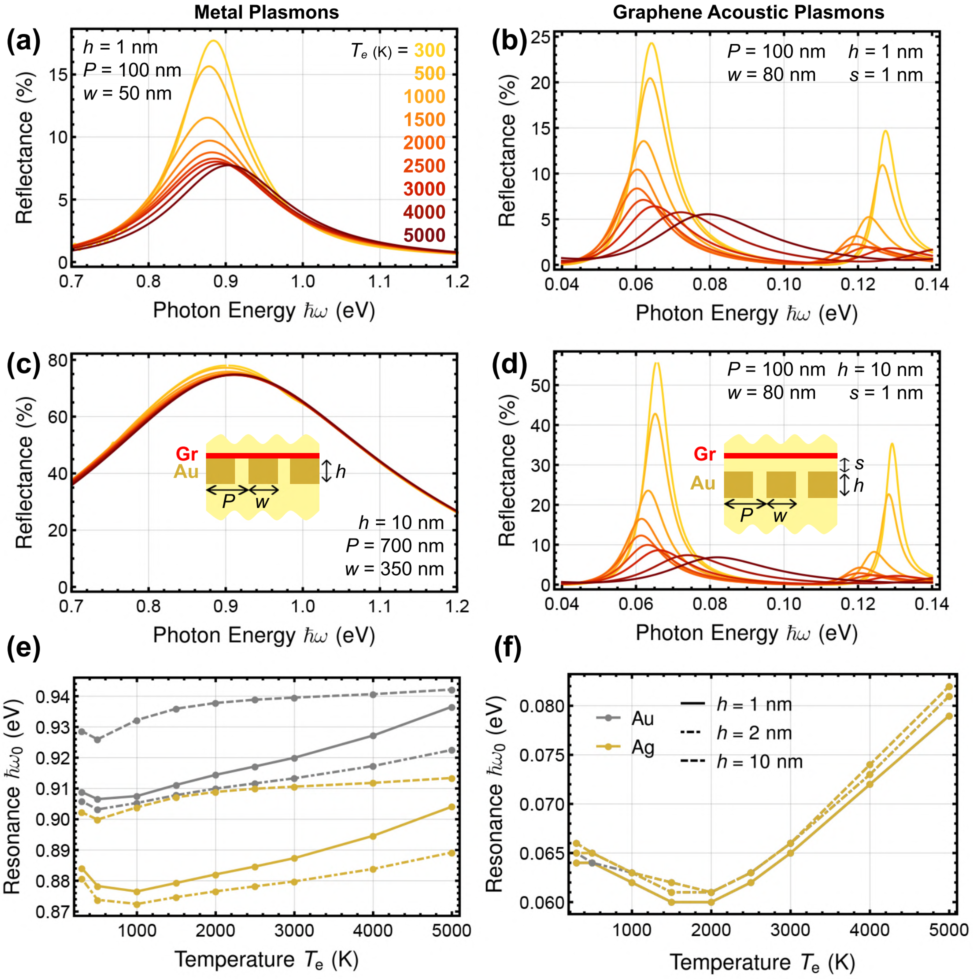

FIG. S4: Thermal modulation of plasmons in graphene/structured-metal hybrid systems. (a-d) Variation of the reflection spectra of graphene/gold gratings in the (a,c) NIR and (b,d) far/mid-IR plasmonic regions for different graphene electron temperatures T_e; the gold thickness is 1 nm in (a,b) and 10 nm in (c,d). (e,f) Resonance plasmon energy $\hbar\omega_0$ (extracted from panels (a-d) and Fig. 4(a-d) of the main text) as a function of graphene electron temperature for the geometrical parameters indicated in the corresponding Figs. 4(e,f) in the main text. In (a,c,e), plasmons are metal-like and graphene is doped to $E_F = 0.55$ eV. In (b,d,f), plasmons are acoustic and graphene is doped to $E_F = 0.4$ eV. All structures are embedded in an $\epsilon = 2$ dielectric.

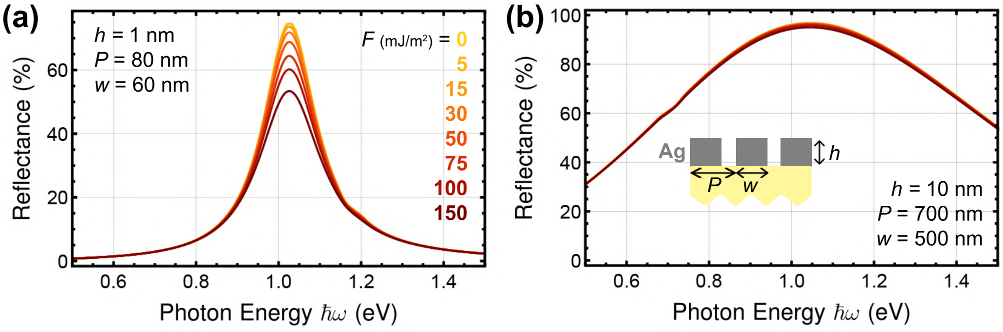

FIG. S5: Thermal modulation of silver plasmons. We show the variation of the reflection spectra in silver gratings sitting on an $\epsilon = 2$ substrate for different optical pump fluences at 785 nm wavelength. The silver thickness is 1 nm in (a) and 10 nm in (b).
